# Monkeypox: An Emerging Disease

**DOI:** 10.7759/cureus.29393

**Published:** 2022-09-21

**Authors:** Kapil Sharma, Shivani Akre, Swarupa Chakole, Mayur B Wanjari

**Affiliations:** 1 Medicine, Jawaharlal Nehru Medical College, Datta Meghe Institute of Medical Sciences, Wardha, IND; 2 Community Medicine, Jawaharlal Nehru Medical College, Datta Meghe Institute of Medical Sciences, Wardha, IND; 3 Research, Jawaharlal Nehru Medical College, Datta Meghe Institute of Medical Sciences, Wardha, IND

**Keywords:** endemic, pcr, self-limiting, lymphadenopathy, smallpox

## Abstract

Until the month of April 2022, cases of monkeypox virus infection in humans were hardly documented outside of the endemic African regions. There are cases now throughout the world. Infected exotic pets have taken the monkeypox virus out of Africa. Following the universal eradication of smallpox in the 1970s, occurrences of monkeypox have attracted attention on a global scale. The western hemisphere and European nations are seeing the majority of the monkeypox infections linked to the 2022 epidemic. Numerous groups are working on contact-tracing initiatives, but it is still unclear what started this outbreak. The precise cause of monkeypox is uncertain, as the virus's origins have been linked to a number of rodents and small animals. Testing for monkeypox DNA from a patient using polymerase chain reaction (PCR) or viral culture isolation material can both be used to confirm monkeypox infection. Monkeypox is from the family: Poxviridae, subfamily: chordopoxvirinae, genus: orthopoxvirus and species: Monkeypox virus. The DNA virus monkeypox virus (MPXV), which causes the zoonotic illness MPX, or monkeypox, is divided into two genetic clades: The Congo Basin (CB) and the west Africa (WA) clades. Monkeypox's true impact on public health is uncertain.

## Introduction and background

**Figure 1:**
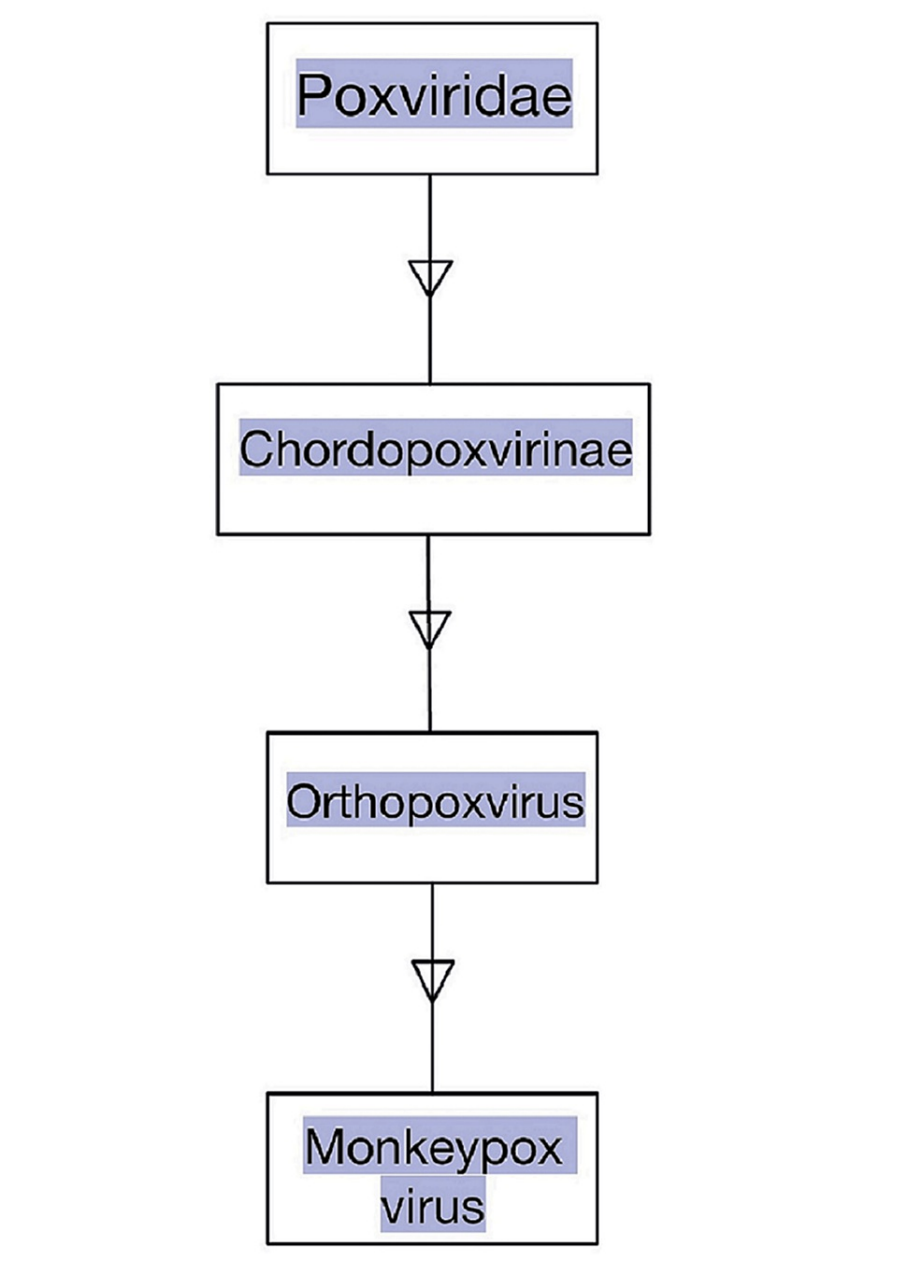
Family, Subfamily and Genus of Monkeypox Virus Image credits: Kapil Sharma

A zoonotic orthopoxvirus called monkeypox (MPX) inadvertently produces smallpox-like illness in humans, but with far lesser fatality. Poxviruses are brick-shaped and have a linear double-stranded DNA genome enclosed in a lipoprotein sheath. The fact that this virus is indigenous to widespread outbreaks across the western hemisphere, as well as western and central Africa which can be connected to the trade of exotic pets and traveling abroad, makes it therapeutically significant [1]. When monkeys were brought from Singapore to a study facility in Denmark and became unwell in 1958, they were first segregated as monkeypox virus [2]. The viral illness was originally noticed in macaque monkeys, thus given the name monkeypox. In 1970, the virus was discovered in a child in the Democratic Republic of the Congo who had been diagnosed with smallpox, being the first known instance in a human [3]. Additionally, given that rural Africa is where monkeypox cases are most common, it is possible that underreporting could result in an underestimate of the pathogen's potential impact (Figure 1) [4-6].

## Review

Pathogenesis and causes

**Figure 2:**
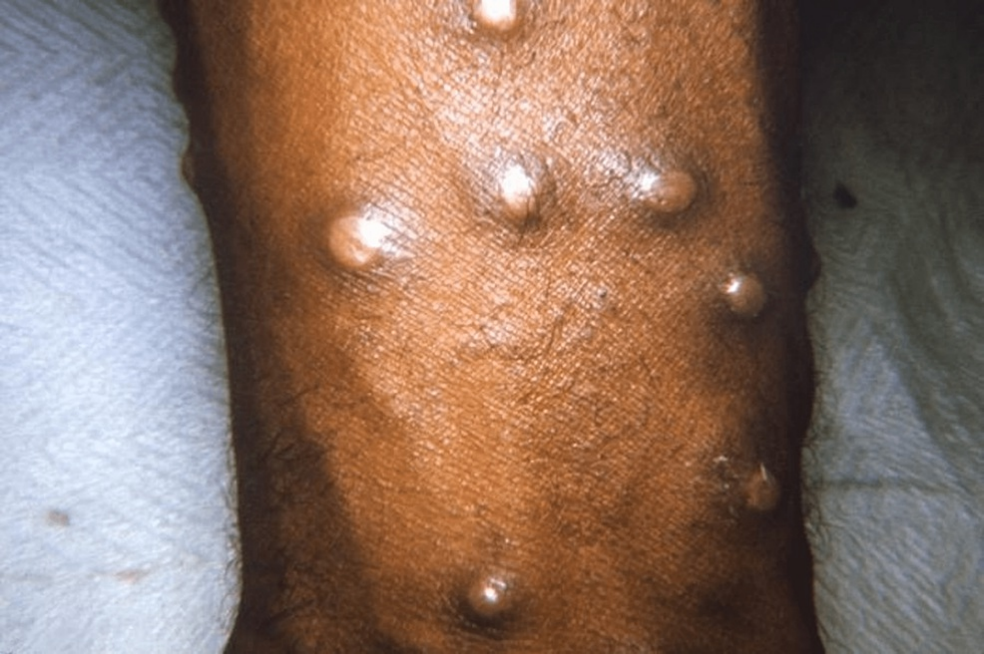
Maculopapular Lesions on Arm Open access journal under a CC-BY license Contributed by Dr. John Noble, Jr., The Centers for Disease Control and Prevention (CDC)

Animals can contract the viral illness known as "monkeypox", primarily some primates, including humans, rodents, and monkeys, but has milder symptoms than smallpox. Smallpox and cowpox viruses are in the same family as the monkeypox virus. Humans can contract the monkeypox virus from sick animals by getting bitten by them or directly coming into contact with their bodily fluids. Extended close contact, typically between family members, can also cause person-to-person transmission. Exposure to respiratory droplets may also be a means of transmission [7]. The genomes of poxviruses contain all required replication, transcription, assembly, and egress proteins [8]. According to observations, monkeypox has an enclosed, pleomorphic core with lateral bodies. Monkeypox is less deadly than smallpox, 10% or less of the total cases result in death [9]. The monkeypox virus has two distinct genetic clades, the central African (Congo Basin) clade and the west African clade [10]. Men having sex with men (MSM) is an important cause of the high rate among the male gender. In the past, more serious sickness has historically been brought on by the Congo Basin clade and is supposedly more virulent than the other genetic group (Figure 2) [11].

The west African clade's open reading frames had deletions and fragmentations that reduced its pathogenicity. In human cells made from patients who had previously contracted monkeypox, central African monkeypox suppresses T-cell receptor-mediated T-cell activation, preventing the generation of inflammatory cytokines [11,12]. The central African strain preferentially inhibits the host's defense mechanisms, particularly the host's apoptosis [11]. In the presence of monkeypox with a modest viral burden, responses to cytokines mediated by T-cells were 80% reduced, indicating that it produces a modulator that decreases host T-cell responses might be produced by monkeypox [13]. The monkeypox virus might be protected by the smallpox vaccination. Monkeypox instances increased once the smallpox vaccination was no longer administered [12]. Since west African variants of the monkeypox virus lack the complement enzyme inhibitor, this immune-modulating component has been linked to the central African strains' higher virulence [14,15].

After viral penetration, the monkeypox virus reproduces at the entry site by any channel (oropharynx, nasopharynx, or intradermal), then spreads to nearby lymph nodes, causing seeding of other organs with viruses and viral dissemination which are caused by viremia. This is the incubation stage that can last up to 21 days and normally lasts 7 to 14 days [16-18]. Before lesions are visible, there are 1-2 days of prodromal signs including lymphadenopathy and fever caused by secondary viremia. Patients who are sick now could be spreadable. Skin lesions develop from oropharyngeal lesions. By the time lesions start to form, serum antibodies are frequently detected [19].

History to diagnose monkeypox

A differential diagnosis can be made using historical markers of monkeypox infection, such as recently traveling to areas where it is endemic, contact with native wild creatures that have been imported, and caring for an infected animal or human, but clinical symptoms are crucial. Close contact with respiratory secretions and infected person's skin sores or contaminated clothing and bedding can spread diseases from one person to other [20]. Veterinarians and other animal workers are among the groups of persons who may be exposed to the monkeypox virus. Congenital monkeypox can also result via vertical transmission of the virus from a mother to a fetus. It is yet unknown if monkeypox may be transferred sexually, despite the fact that a well-known risk factor for transmission is physical touch [21]. 

Clinical features

The symptoms of MPX, which have 4-21 days of incubation, with symptoms ranging from fever to respiratory distress, are identical to those of smallpox with the exception of lymphadenopathy. Exhibiting symptoms for two to four weeks, monkeypox is a self-limiting illness (4-14 days) [20]. Initial warning signals and symptoms are frequently nonspecific. Fever, headache, myalgia, tiredness, and lymphadenopathy are some of the first symptoms. Following exposure, a vesiculopustular rash appears 12 to 16 days later over the face and trunk before spreading centrifugally to different body parts, such as the palms and soles [20-22]. Rash lesions develop morphologically in several phases, including macular, papular, vesicular, and pustular lesions [23]. Lesions range in size from 2 to 10 mm, are hard, and undergo synchronous change. The pustules eventually develop crusts, which, after one to two weeks, desquamate. With sensitive maxillary, cervical, and inguinal lymphadenopathy which is a prominent characteristic [11,24]. The development of lymphadenopathy indicates that the immune system may be responding more strongly and recognizing the monkeypox virus. Once all the crusts have disappeared, the patient is no longer considered infectious [25]. The polymerase chain reaction (PCR) test can confirm a case of monkeypox that has been suspected. Given the similarity between human monkeypox infection and smallpox, it may be possible to select patients for further testing using the "Acute, Generalized Vesicular or Pustular Rash Illness Protocol" developed by the CDC (The Centers for Disease Control and Prevention), which includes lymphadenopathy in addition to the necessary primary criteria [11]. If the virus spreads to an individual who is immunocompromised, there might be serious illness or even death [25].

Treatment modalities

Fortunately, monkeypox infections often have a clinical course that is modest and self-limiting. Consequently, it rarely needs specialized therapy, and instead, treatment is supportive. There are currently no known cures for monkeypox, although smallpox research shows that the treatment options for monkeypox include the vaccinia vaccine, cidofovir, tecovirimat, and vaccinia immune globulin (VIG), however, there is little evidence to back this up [26]. The World Health Organization states the European Medicines Agency (EMA) granted tecovirimat monkeypox licensure in 2022 after it was developed for smallpox. Both oral (200 mg capsule) and injectable versions of tecovirimat are offered. It can be used for monkeypox in the US, based on data from CDC. Fever-reducing medications and painkillers, and medicines for bacterial infections that come later are all examples of supportive care. However, certain individuals could need a particular kind of care. For patients with serious sickness, individualized treatment could be required. Patients with impaired immune systems, expectant mothers, and youngsters may also need specialized care [10]. Additionally, the FDA-approved antiviral drugs cidofovir or brincidofovir, used to treat the cytomegalovirus (CMV) and smallpox, respectively, may be employed. An immunoglobulin called vaccinia immune globulin intravenous (VIGIV) is used to treat vaccinia vaccination adverse effects. Its use as a therapy for monkeypox illness is permitted by the US CDC under an enhanced access policy [10].

Prognosis of the disease

Most cases of monkeypox are self-limiting, exhibiting symptoms for two to four weeks. However, some patients could have serious illnesses. Serious cases of monkeypox are more common in children, which are correlated with the patient's health state, the degree of viral exposure, and the kind of consequences [27,28]. The results might be worse if immunological deficits were present. Few hospitalizations have been noted during the global epidemic of 2022, and the majority were done with the intention to isolate the patient [29].

Modalities to prevent monkeypox disease

**Figure 3:**
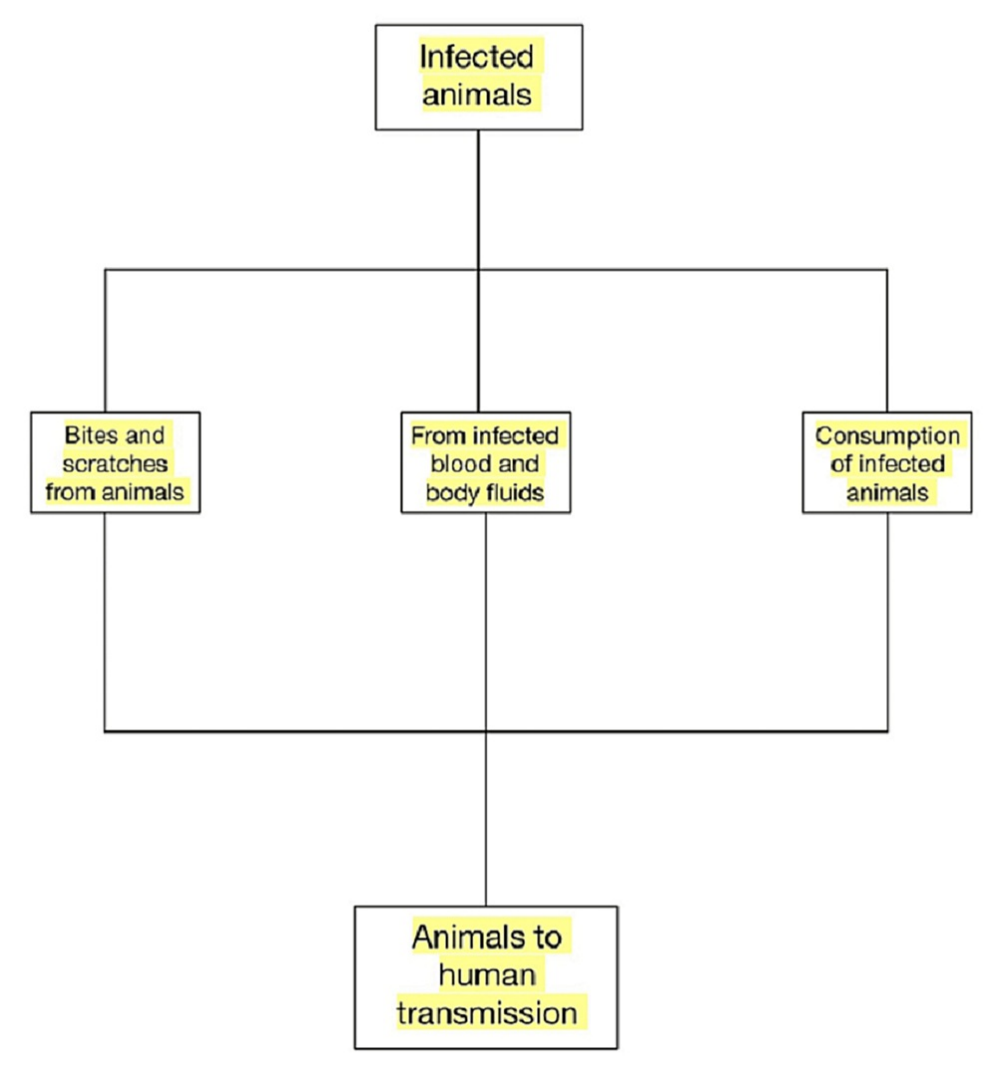
Route of Transmission of Monkeypox From Animal to Human Image credits: Kapil Sharma

In order to prevent the transmission of monkeypox in locations where it is prevalent, according to the CDC direct sexual contact oral, anal, and vaginal sex or touching the genitals of a person and direct contact with blood with monkeypox need to be inhibited. For the public to become more informed, massive health education activities are needed, provide instructions on how to handle possible animal reservoir species (gloves, protective clothes, surgical masks), and warn people to stay away from afflicted people. The prevention of human-to-human transmission in healthcare depends on infection control techniques. Improved isolation procedures and nursing techniques call for training as well as sufficient resources in the form of staff and facilities. Health professionals and anybody treating or coming in contact with monkeypox patients should get immunized. According to estimates, receiving a smallpox immunization offers 85% cross-protection against monkeypox infection [30]. Living in a forest or a newly cleared woodland, not getting vaccinated against smallpox, eating or handling deceased animals, such as monkeys or bushmeat, and slumbering on the ground (in endemic areas) are dangers for viral zoonotic transmission [22]. The patient should be put in a private room or a negative air pressure isolation chamber as soon as possible if they are suspected of having the condition. When entering the patient's room, medical professionals should put on personal protective equipment (PPE), which includes a gown, gloves, eye protection, and an N95 or higher respirator (Figure 3).

Differential diagnosis

The most typical alternative medical diagnosis is chickenpox. In contrast to chickenpox, which has a brief prodromal time, centrifugal rash distribution, no lymphadenopathy, and a rapid rate of rash spread, monkeypox has a long prodromal phase, centrifugal rash distribution, and lymphadenopathy [30]. Other differential diagnoses include measles, infected scabies, drug eruptions, molluscum contagiosum, secondary syphilis, and hand, foot, and mouth disease.

## Conclusions

The monkeypox virus causes the zoonosis known as human monkeypox. Monkeypox virus is an orthopoxvirus related to smallpox, first noted in 1970 in central Africa. Humans have a fever, headache, general malaise and weariness, enlarged lymph nodes, and other symptoms two weeks after infection. A rash of raised pimples develops on the face and torso a few days later. The sickness gradually takes its course in two to four weeks and eventually crusts and peels off. Transmission may occur through direct contact with bodily fluids, skin lesions, respiratory droplets of infected animals, or indirect contact with contaminated fomites. Contact with an infected person's skin sores or respiratory secretions, or human-to-human transmission can also occur as a result of contaminated clothing and bedding. However, for transmission, prolonged face-to-face contact is frequently required via respiratory droplet particles increasing the vulnerability of medical professionals, family members, and any close associates of active cases. Given alleged flaws in disease reporting and confirmation, precise prevalence and incidence are challenging to establish. However, after the end of the regular smallpox immunization program, these measures have grown. Only symptoms are addressed during treatment. By separating patients and maintaining good cleanliness around them, outbreaks are controlled. It is crucial that local and international authorities raise awareness and take action to prevent the virus spread in the community. Careful collaboration between public health authorities, doctors, and the community will be necessary to contain this expanding international pandemic.
